# Application of Transthoracic and Endobronchial Elastography—A Systematic Review

**DOI:** 10.3390/cancers18020190

**Published:** 2026-01-07

**Authors:** Christian Kildegaard, Rune W. Nielsen, Christian B. Laursen, Ariella Denize Nielsen, Amanda D. Juul, Tai Joon An, Dinesh Addala, Casper Falster

**Affiliations:** 1 Department of Respiratory Medicine, Odense University Hospital, 5000 Odense, Denmark; 2 Odense Respiratory Research Unit (ODIN), Department of Clinical Research, University of Southern Denmark, 5000 Odense, Denmark; 3 Department of Cardiology, Odense University Hospital, 5000 Odense, Denmark; 4 Centre for Advanced Lung Cancer Diagnostics (CALU), Odense University Hospital, 5000 Odense, Denmark; 5 Department of Infectious Medicine, Odense University Hospital, 5000 Odense, Denmark; 6 Department of Internal Medicine, Odense University Hospital, 5700 Svendborg, Denmark; 7 Division of Pulmonary and Critical Care Medicine, Department of Internal Medicine, Yeouido St. Mary’s Hospital, College of Medicine, The Catholic University of Korea, Seoul 07345, Republic of Korea; 8 Department of Respiratory Medicine, Churchill Hospital, Oxford University Hospitals NHS Foundation Trust, Oxford OX3 7LE, UK

**Keywords:** elastography, thoracic ultrasound, endobronchial ultrasound, cancer, pleura, interstitial lung disease, biopsy

## Abstract

**Introduction:** Ultrasound elastography is increasingly used across medical imaging, yet its role in thoracic disease remains poorly defined. While both transthoracic ultrasonography (TUS) and endobronchial ultrasound (EBUS) offer real-time assessment of pleural and pulmonary structures, the diagnostic and clinical value of elastography in this context remains uncertain. **Materials and Method:** A systematic search of MEDLINE, EMBASE, and the Cochrane Library was conducted according to PRISMA guidelines (April 2023; updated January 2025). Original studies evaluating transthoracic or endobronchial elastography for pleural or pulmonary conditions were included. Data extraction and quality assessment were performed independently by three reviewers, with QUADAS-2 used to evaluate risk of bias. **Results:** Thirty studies met inclusion criteria. Twenty-eight evaluated TUS elastography and two examined EBUS. Shear wave elastography was most frequently applied, particularly for differentiating malignant from benign pleural effusion or subpleural lesions. Surface wave elastography demonstrated consistently higher stiffness values in patients with interstitial lung disease compared with healthy controls, correlating with radiological and functional disease severity. Elastography-guided pleural biopsy improved diagnostic yield compared with conventional ultrasound-guided biopsy. Overall, substantial methodological variation existed among scanning techniques, elastography modalities, reporting methods, and diagnostic thresholds, limiting cross-study comparison. **Conclusions:** Ultrasound elastography shows promise for evaluating pleural effusion and pulmonary lesions, procedural guidance, and interstitial lung disease possibly improving diagnostic possibilities with bedside evaluation and reducing patient exposure to radiation. However, methodological variation and limited high-quality evidence preclude clinical implementation. Standardized acquisition protocols and multicentre validation studies are necessary to define its diagnostic utility in thoracic imaging.

## Simple Summary

Ultrasound elastography is increasingly applied in thoracic imaging, but its clinical role remains unclear. This systematic review of 30 studies found that most research focused on transthoracic applications, particularly shear wave elastography for distinguishing malignant from benign pleural effusions or subpleural lesions, and surface wave elastography for assessing interstitial lung disease. However, substantial variation in methods, ultrasound techniques, and diagnostic thresholds restricts comparison across studies. Overall, elastography shows potential value in thoracic disease evaluation and procedural guidance, but standardized protocols and larger validation studies are needed before widespread clinical adoption.

## 1. Introduction

Ultrasound is a well-established modality for investigating thoracic diseases. Thoracic ultrasound (TUS) can be performed at bedside, is non-invasive, and reduces patient exposure to ionizing radiation and, in some cases, contrast agents [1]. Several features can guide the diagnostic and therapeutic approach toward conditions such as pneumothorax, pleural effusion, parietal pleural abnormalities, and lung parenchymal pathology [2,3,4]. While several findings such as the pleural line, pleural effusion, B-lines, and consolidations are of clinical value, additional information when performing ultrasound examination on underlying disease could prove useful to risk stratification and diagnostic guidance [5,6]. Endobronchial ultrasound (EBUS) has transformed modern bronchoscopic practice, offering a relatively safe and minimally invasive modality for evaluating a wide range of intrathoracic diseases. This technology has expanded diagnostic and therapeutic options, particularly in the management of intrathoracic lymphadenopathy and pulmonary lesions [7]. While these modalities offer utility in detecting pleural and parenchymal abnormalities, they lack the ability to differentiate between different underlying pathologies giving rise to the same ultrasonographic presentation.

Elastography is based on the principle of tissue elasticity, and a tissue deformation by an external force can be measured. Depending on the modality in use, the deformation can be expressed as either longitudinal or shear waves [8,9,10]. Application is either determined as strain or shear wave measurements, but studies have also explored the use of surface wave elastography, especially in terms of assessing interstitial lung disease (ILD) [11,12].

A comprehensive guideline and recommendation for the use of elastography on several organs has been published by the European Federation of Societies for Ultrasound in Medicine and Biology (EFSUMB) [13,14]. The validation of elastography’s applicability to assess certain organs, e.g., liver fibrosis, focal breast lesion, and lymph node, is well established, but no current recommendations are available to direct the assessment of thoracic diseases. A recent systematic review by Vargas et al. in 2024 reviewed the current evidence regarding pleural effusion, pulmonary lesions, and ILD, which indicated a promising sign of implementation of ultrasound elastography in pleuropulmonary evaluation; however, studies lack standardization [15]. Although comprehensive, the review only identified 613 papers for screening and did not cover endobronchial elastography. Furthermore, as a novel ultrasonographic modality in respiratory medicine, new data on the evaluation of thoracic conditions are frequently published.

As such, a systematic review is warranted, and the aim of this study was to conduct a systematic literature search on transthoracic and endobronchial elastography and provide an overview of the current literature along with its capabilities to assess pulmonary and pleural conditions.

## 2. Materials and Methods

Prior to the literature search and data extraction, the project was registered at The International Database of Prospectively Registered Systematic Review of Health Related Outcomes (PROSPERO) (ID CRD42023420222). The work of this systematic review was conducted in accordance with Preferred Reporting Items for Systematic Reviews and Meta-Analysis (PRISMA) to ensure appropriate reporting (Supplementary Table S1).

### 2.1. Eligibility Criteria

The following eligibility criteria were applied to ensure clinical relevance and methodological consistency.

Inclusion criteria

- Ultrasound elastography in all iterations, investigating thoracic conditions.

Exclusion criteria

- Animal studies;
- Phantom studies;
- Evaluation for lymph nodes, costal or intercostal structures;
- Conference abstract or highlights report.

### 2.2. Search Strategy

The search strategy was developed in collaboration with a professional research librarian. The Patient-Intervention-Comparison-Outcome (PICO) outline was developed as shown:

- *Population:* All humans who had their thorax assessed with any type of elastography.
- *Index test:* Transthoracic or endoscopic ultrasound elastography.
- *Reference test:* Diagnostic tests considered gold standards encompass, but were not limited to, pathology or cytology by transthoracic or endoscopic biopsy, microbiological studies or clinical follow-up elastography examination. For studies aiming at establishing reference values in healthy subjects, no reference standard was required.
- *Diagnosis of interest:* Any pathology or description of physiological conditions of the thorax.

From this, the following search string was constructed:

Block 1: Lung
Lung [MeSH] OR Pulm* OR Lung* OR Pneu* OR Pleura [MeSH] OR Pleur*
Block 2: Pulmonary embolism
Elasticity Imaging Techniques [MeSH] OR Shearwav* OR Elastograph* OR Fibroscan*

A primary systematic search was conducted on the 21 April 2023 of MEDLINE, EMBASE, and Cochrane Library databases. Following systematic inclusion and exclusion of eligible articles, a second search was performed on the 15 January 2025 to ensure newly published relevant articles were included. No filters (e.g., date of publication or language) were applied. After removing duplicates, CK, RWN, and ADN independently evaluated articles based on title and abstract. Subsequent full text screening of articles deemed eligible by title and abstract was performed by CK and RWN. In case of disagreement, a third investigator, CF, decided if the article was eligible. A snowballing search of all included studies’ references were performed as a final search for eligible articles. Screening was performed in Covidence (Veritas Health Innovation Ltd., Melbourne, Australia). Included articles were managed with the use of EndNote 20 (Clarivate Analytics, Philadelphia, PA, USA).

### 2.3. Grouping of Included Studies

The included studies were categorized as transthoracic or endobronchial ultrasound elastography. Studies on thoracic ultrasound were further subcategorized into five groups:

(A) Pleural effusion;
(B) Pulmonary consolidations;
(C) Interstitial lung disease;
(D) Procedural guidance;
(E) Other.

### 2.4. Risk of Bias and Quality Assessment

Assessment of quality and bias was performed on all included articles by CK and RWN using the Quality Assessment of Diagnostic Accuracy Studies-2 tool, QUADAS-2 [16].

## 3. Results

**Figure 1:**
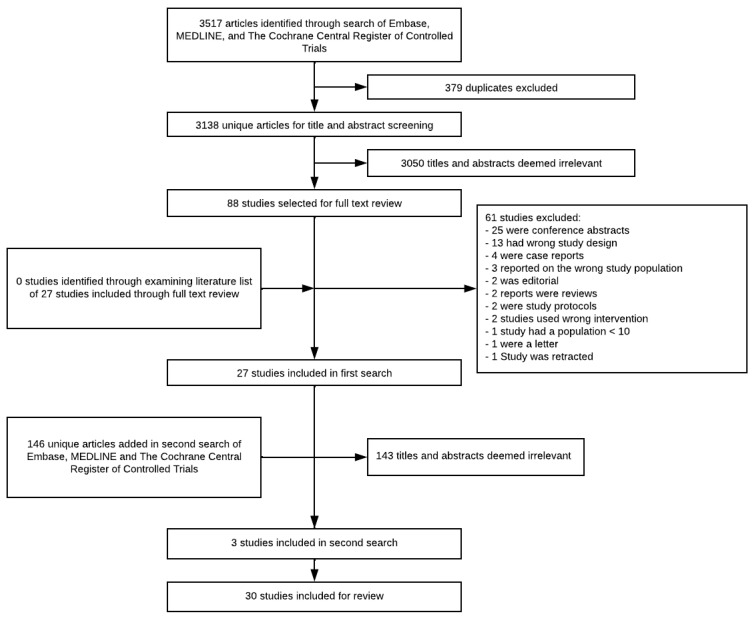
PRISMA flowchart of search and exclusion of articles.

The systematic literature search generated a total of 3663 articles. Duplicates were identified in 389 instances, and 3193 articles were deemed irrelevant when screening title and abstract. This resulted in full text screening of 97 studies, of which 30 were eligible for inclusion. Of the 30 included articles, three were identified in the secondary search. Evaluation of the reference lists of included articles yielded no additional relevant articles. A summary of the search construction is available in Figure 1.

Of the 30 papers included, 28 reported on the use of TUS, while two investigated EBUS. Of the 28 papers investigating TUS elastography, eleven examined pulmonary lesions. Pulmonary effusion and ILD were investigated in four and seven papers, respectively.

### 3.1. TUS

- Pleural Effusion

Four papers investigated the utility of elastography in relation to pleural effusion from three different countries [17,18,19,20]. All studies applied Shear Wave Elastography (SWE). Two studies investigated the ability of SWE in differentiating malignant from benign effusions. The others assessed the ability of distinguishing transudative from exudative effusion or expandable from non-expandable lung (Table 1 and Table 2).

B. Pulmonary Lesions

Eleven studies reported on pulmonary lesions assessed by TUS elastography [22,23,24,25,26,27,28,29,30,31,32]. These studies were published between 2013 and 2022 from five different countries. Six studies applied SWE as either two dimensional (2D-SWE) or point (pSWE) (Table 3). Eight studies investigated the ability to differentiate between malignant and benign subpleural lesions (Table 3 and Table 4). Additionally, pulmonary consolidation was also measured beneath pleural effusion by Petersen et al. and Nielsen et al. [19,20].

C. Interstitial Lung Disease

Seven papers investigated the use of elastography in assessment of ILD (Table 5) [12,33,34,35,36,37,38]. All articles except one are case-control studies, describing utility of surface wave elastography. The majority of compared elastography findings to high-resolution computed tomography (HRCT), pulmonary function test (PFT), and clinical assessment.

Across the reviewed studies, ultrasound elastography consistently demonstrated higher stiffness values in patients with ILD compared with healthy controls. All studies using surface wave elastography at 100–200 Hz reported significantly elevated wave velocities in ILD. The modality utilizes measurements at the pleural line movements induced by external shaker and recorded with B-mode. Zhang et al. confirmed significant differences across six lung zones (*p* < 0.0001) [34,35,36,37]. Clay et al. reported that surface wave elastography velocities correlated with radiological fibrosis severity (Area Under the Curve [AUC] = 0.94), while Zhou et al. identified an optimal diagnostic cut-off of 5.47 m/s at 200 Hz, yielding 92% sensitivity and 89% specificity [12,37]. In an independent study, Huang et al. reported higher pleural line stiffness in Connective Tissue Disease (CTD)–associated ILD using 2D-SWE [38].

D. Procedural Guidance

One study by Deng et al. investigated the use of elastography as procedural guidance for biopsy. A randomized controlled trial with 1:1 allocation was performed, and a total of 228 patients were included in the analysis. Patient were randomized to either thoracic ultrasound-guided or elastography-guided pleural biopsy. A cut-off value for target biopsy was set at a minimum of 47.25 kPa, indicating malignant pleural thickening. A significant greater sensitivity (80.49% vs. 50.00%, *p* = 0.007), and diagnostic yield (87.83% vs. 76.99%, *p* = 0.032) was observed with elastography-guided biopsy (Table 6).

E. Other

Five papers reported on conditions not encompassed by Sections A–D, exploring the elastographic value in the examination of chronic obstructive pulmonary disease (COPD), pneumothorax, and pulmonary edema (Table 7). Half of these articles (3/6) examined strain elastography (SE), while SWE and surface wave elastography were investigated in two papers each.

Nouvenne et al. demonstrated in a feasibility study that SE values were higher in COPD than healthy smokers. Furthermore, examination of posterior basal zones demonstrated a valuable site for distinguishing between healthy non-smokers and COPD/smokers (AUC = 0.846, 95% confidence interval [CI] 0.73–0.93, *p* < 0.001) [42]. In patients undergoing general anesthesia, Girard et al. demonstrated a correlation between ventilation and pleural strain measured by SE and SWE. An excellent intraobserver agreement was observed, although interobserver agreement was moderate to good [40]. Among 30 patients suspected of pneumothorax, Bandelli et al. could confirm the lung point using SE in all patients, indicating the feasibility of clinical integration [41]. One healthy subject was investigated by Zhang et al. in a feasibility study of surface wave elastography, demonstrating the feasibility of surface wave elastography and that increased surface wave speeds measured corresponded to the amplified frequency of external mechanical vibration [44]. The basic principles of surface wave elastography are further examined in papers of ILD patients (Section C). Finally, Wiley et al. demonstrated on 14 patients a significant decrease in surface wave speed from admission to 1–2 days after diuretic therapy [43].

### 3.2. EBUS

B. Pulmonary lesions

Two papers reported the use of elastography on pulmonary lesions during endobronchial investigation (Table 8) [29,30]. Diagnostic performance was moderate to good, with histopathological outcome as the diagnostic reference. Using a 4-point scoring system, He et al. demonstrated that malignant lesions exhibited a significantly higher score compared to non-malignant lesions. With an AUC of 0.793, an optimal cut-off point was set to be 2.5, with a sensitivity and specificity of 72.2% and 76.2%, respectively. The elastography grading score was superior compared to all other ultrasound modalities [29]. Zhi et al. performed elastography during EBUS and categorized it according to a 1–5 scale, with a dichotomous outcome of 1–3 classified as benign, while 4–5 was considered malignant. A cut-off of 6.5 was found optimal to ensure an AUC of 0.692, with a corresponding sensitivity and specificity of 83.5% and 52.6%, respectively. The intra- and interobserver agreement was found to be 0.951 and 0.886, respectively [30].

### 3.3. Risk of Bias and Quality Assessment

The overall risk of bias in studies included in this systematic review was deemed high, with unclear perspectives reported and the majority of thresholds not reported in advance (Supplementary Figure S1). Assessment of applicability was at a moderate-to-high level, but concerns regarding patient flow and patient selection were observed (Supplementary Figure S2).

## 4. Discussion

Based on the current evidence identified as part of this review, elastography seems to harbor potential in regard to examining pulmonary conditions. However, contemporary evidence does not allow definite assessment of the clinical utility and optimal implementation of this modality.

### 4.1. Clinical Implication

In the case of pleural effusion, SWE consistently demonstrated the capability to quantify biomechanical differences relevant to differentiating effusion etiologies. Studies attempting differentiation between malignant and benign effusions, as well as transudative versus exudative fluid, reported discriminative stiffness values that support potential clinical integration [17,18,19]. In cases in which thoracentesis or diagnostic tap is not possible, elastography could possibly provide additional clinical information to assess possible malignancy. Similarly, elastography-based assessment of an expandable versus non-expandable lung showed feasibility [20]. However, despite encouraging findings, the variability in acquisition conditions, ranging from controlled inpatient settings to emergency care environments, limits the generalizability of reported diagnostic thresholds.

Elastographic characterization of subpleural pulmonary lesions constitutes the most extensively studied domain. Across studies, both SE and SWE demonstrated the ability to distinguish malignant from benign lesions with moderate-to-good diagnostic performance [22,23,24,25,26,27,28,29,30,31,32]. Based on current data, elastographic assessment would not be a stand-alone single diagnostic test to rule in/out pulmonary malignancy. On the contrary, elastographic measurements could prove valuable in collected imaging assessment and could improve diagnostic pathways. More recent transitions from SE toward quantitative SWE have improved reproducibility and may offer more stable cut-off values than earlier qualitative approaches [22,29,30]. Nevertheless, the heterogeneity in ultrasound transducer types, patient positioning, breath-hold requirements, and Region-of-Interest (ROI) definition complicates the interpretation of pooled diagnostic accuracy, as highlighted by Kuo et al. [22]. Despite these limitations, the consistency of higher stiffness values in malignant lesions across studies suggests a meaningful diagnostic signal.

Only two studies have been published that have assessed EBUS elastography. The methodologies across EBUS elastography were innovative but methodologically diverse [21,45]. Diagnostic performance was moderate to good, showing promising steps. A difference in scoring model was observed between the two studies, but a high intra- and interobserver agreement is suggestive that EBUS elastography may improve identification of malignant lesions [21]. As to subpleural lesions, EBUS elastography could serve as a complementary modality to further enhance diagnostic yield. The area is understudied compared to transthoracic evaluation as elastography technology is still evolving and not yet widely integrated into standard bronchoscopic platforms. Comparative trials against existing modalities, assessment of learning curves, and evaluation of clinical impact on biopsy decision-making and patient outcomes would further clarify its utility and accelerate transition toward broader clinical adoption.

Evidence for elastographic assessment of ILD is primarily derived from lung surface wave elastography [12,33,34,35,36,37]. These findings indicate that elastography may provide a non-invasive adjunct for detecting fibrotic lung changes and potentially monitoring disease progression, ultimately saving patients from radiation with repetitive HRCTs. Although several studies indicate a correlation between surface wave measurement and ILD, the elastographic protocol is relatively new, not recognized in other settings, and inflicts procedural difficulties and observer variation

One study, a randomized controlled trial, evaluated elastography for procedural guidance. In this study, elastography-guided pleural biopsy significantly improved sensitivity and diagnostic yield compared with conventional ultrasound, suggesting a possible improvement in the diagnostic procedure [39]. Implementation of elastography could possibly secure an optimal diagnostic pathway for patients and reduce possible re-biopsy procedures, including reduced procedure-related complications. Replication is needed before this technique can be recommended for routine clinical use. Furthermore, the evaluation of elastographic differences in subtype malignant diseases has not been evaluated, which could lead to misinterpretation of optimal biopsy site if subtypes prove to have diverse properties.

### 4.2. Methodological Concerns

From a methodological perspective, several components are not standardized in current evidence published. Across all study categories, methodological variability represents the major constraint limiting evidence synthesis and clinical extrapolation.

In general, published studies were of single-center setting, limiting the external generalizability, and of small sample size with no prior power estimate. The elastographic acquisition protocols included variation in breath-hold instructions, diverse patient positioning and scanning protocols, with a minority of papers describing the ROI placement. Several different modalities of elastography have been investigated, emphasizing the wide range of elastographic methods but without standardization and thus complicating cross-study comparison [12,17,18,20,24,25,26,32,34,44]. In the evaluation of diagnostic methods, knowledge of intra- and interobserver variation is important to guide possible clinical implementation. Although high agreement was observed regarding reproducibility for the most part in the studies assessed, observer variability is inconsistently reported [17,18,22,27]. Lastly, as expressed in the risk-of-bias assessment, the concerns regarding patient selection, unclear thresholds, and insufficient reporting all further limit drawing definitive conclusions and future large-scale, standardized studies.

A more standardized framework for future transthoracic elastography studies should include a uniform ROI definition within the stiffest homogeneous lesion area, with ROI size reported and scaled-to-lesion dimensions. Breath-hold instructions—preferably at end-expiration—should be applied to limit motion artifact, alongside consistent patient positioning, probe orientation, and machine settings. Quantitative outputs should use clearly defined metrics (e.g., mean vs. maximum values, kPa vs. m/s), and qualitative scoring systems should be predefined. The methodology should be altered according to modality and target disease evaluated.

### 4.3. Limitations

While this systematic review summarizes current evidence on transthoracic and endobronchial elastography, several review-related limitations should be acknowledged. Despite a broad search strategy, some relevant studies—especially unpublished work—may have been missed. Substantial heterogeneity in study design, elastography technique, and reported outcomes prevented meta-analysis and limited the review to a qualitative synthesis. Screening and data extraction were conducted by a small team, introducing potential selection or extraction bias despite adjudication. Moreover, the long inclusion period spans major developments in elastography technology, complicating comparisons across studies. Finally, exclusion of conference abstracts may have introduced publication bias and have broadened the evidence base by capturing early or unpublished data, potentially affecting effect estimates or revealing less favorable findings. However, the variable rigor and higher risk of bias typical of gray sources might also have reduced overall certainty, leading to more cautious conclusions.

## 5. Conclusions

Elastography in different types of modalities shows some promise in examining pulmonary conditions. However, the collective evidence is characterized by notable methodological variability, precluding meaningful syntheses of the results, as well as significant risk of bias across multiple domains. Consequently, no overall conclusions on the clinical utility of elastography can be drawn. Adequately designed studies with standardized protocols and cut-off values are warranted to assess reproducibility, diagnostic performance, and optimal scanning protocols.

## Figures and Tables

**Table 1:** Studies regarding pleural effusion.

Author	Year	Country	Elastography Method	Study Design	Center	Inclusion Period	Sample Size
Jiang et al. [17]	2018	China	SWE	Diagnostic Accuracy	Single	October 2012–October 2017	130
Nielsen et al. [19]	2025	Denmark	SWE	Diagnostic Accuracy	Single	September 2021–April 2022	27
Ozgokce et al. [18]	2018	Turkey	SWE	Diagnostic Accuracy	Single	June 2016–January 2018	60
Petersen et al. [20]	2025	Denmark	SWE	Diagnostic Accuracy	Single	July 2019–August 2021	98

Abbreviation: SWE = shear wave elastography.

**Table 2:** Results from studies regarding pleural effusion.

Author	Diagnosis	Cut-Off Value	Sensitivity (%)	Specificity (%)	Interobserver Agreement	Intraobserver Agreement
Jiang et al. [17]	Malignant/Benign	47.25 kPa	83.64 (70.70–91.80)	90.67 (81.15–95.85)	SWE-mean: 0.976 SWE-max: 0.581	NR
Nielsen et al. [19]	Malignant/Benign	1.68 m/s	60 (15–95)	55 [21] (32–76)	NR	NR
Ozgokce et al. [18]	Transudative/Exudative	2.52 m/s	76.5%	91%	NR	>85%
Petersen et al. [20]	Expandable/Non-Expandable Lung	Visceral Pleura = 1.64 m/s		AUC 0.59		
		Pleural Effusion = 1.64 m/s		AUC 0.53		
		Parietal Pleura = 2.54 m/s		AUC 0.57		

Abbreviations: SWE = shear wave elastography; m/s = meters pr. second; AUC = area under the curve.

**Table 3:** Studies regarding pulmonary lesions.

Author	Year	Country	Elastography Method	Study Design	Center	Inclusion Period	Sample Size
Kuo et al. [22]	2021	Taiwan	SWE	Diagnostic Accuracy	Single	January 2016–December 2019	233
Wei et al. [31]	2018	China	pSWE SE ARFI	Diagnostic Accuracy	Single	January 2013–January 2015	91
Sperandeo et al. [30]	2015	Italy	SE	Observational Study	Single	September 2012–February 2013	95
Boccantonda et al. [26]	2021	Italy	SWE SE	Observational Study	NR	January 2018–December 2019	14
Quarato et al. [23]	2022	Italy	SWE	Diagnostic Accuracy	Single	November 2018–December 2015	190
Ozgokce et al.	2018	Turkey	SWE	Diagnostic Accuracy	Single	July 2015–December 2016	33
Wanbin Li et al. [27]	2021	China	SE	Observational Study	Single	March 2019–May 2019	153
Alhyari et al. [24]	2022	Germany	ARFI	Diagnostic Accuracy	Single	April 2020–December 2020	87
Huang et al. [28]	2019	China	SWE	Diagnostic Accuracy	Single	November 2017–October 2018	112
Lim et al. [29]	2016	Taiwan	SE	Experimental Study	Single	December 2011–March 2013	45
Ademitz et al. [24]	2013	Germany	Real-Time Elastography	Case Series	Single	NR	18

Abbreviations: SWE = shear wave elastography; pSWE = point shear wave elastography; SE = strain elastography; ARFI = acoustic radiation force impulse.

**Table 4:** Results from studies regarding pulmonary lesions.

Author	Diagnosis	Cut-Off Value		Sensitivity (%)	Specificity (%)	Interobserver Agreement	Intraobserver Agreement
Kuo et al. [22]	Malignant/Benign	65 kPa		94.9	70.1	NR	90.3
Wei et al. [31]	Malignant/Benign	pSWE	1.951 m/s	70.9	69.4	NR	NR
		SE		No significant difference between malignant and benign (*p* = 0.542)			
		ARFI	≥3	83.6	52.8	NR	NR
Sperandeo et al. [30]	Tumor/Pneumonia	≥4		86.9	99.7	NR	NR
Boccantonda et al. [26]	Malignant/Benign	SWV = 3.6 m/s		AUC 0.792 for the diagnosis of lung malignancy Malignant mean = 5.92 ± 2.8 m/s Benign mean = 3.36 ± 1.20 m/s			
		SE = 2.5		AUC 0.688 for the diagnosis of lung malignancy			
Quarato et al. [23]	Malignant/Benign	No statistical difference in between malignant and benign peripheral lesions					
Ozgokce et al.	Malignant/Benign	2.47 m/s		97.7	97.7	NR	NR
Wanbin Li et al. [27]	Malignant/Benign	NR		Malignant 4.24 ± 0.85	Benign 3.41 ± 0.99	*p* < 0.05	0.73 (95% CI 0.65–0.79)
Alhyari et al. [24]	Malignant/Benign	2.21 m/s		89.7	75.3	NR	NR
Huang et al. [28]	Malignant/Benign	5.85 kPa		81.58	80.78	NR	NR
Lim et al. [29]	Differentiation between lesions	Differentiation between lesions		Necrosis significantly different from atelectasis, consolidation, and tumors	Atelectasis significantly different from consolidation and tumor	Consolidation significantly different from tumor	Primary lung cancer significantly different from pneumonia and metastatic lung cancer
Ademitz et al. [24]	Confirmation of lesion	Confirmation of lesion		NR	100%	NR	NR

Abbreviations: pSWE = point shear wave elastography; SE = strain elastography; ARFI = acoustic radiation force impulse; NR = not reported.

**Table 5:** Studies regarding interstitial lung disease.

Author	Year	Country	Elastography Method	Study Design	Center	Inclusion Period	Sample Size
Zhang et al. [33]	2017	USA	Surface Wave Elastography	Case-control	Single	NR	20
Zhang et al. [35]	2017	USA	Surface Wave Elastography	Case-control	Single	NR	71
Zhang et al. [34]	2019	USA	Surface Wave Elastography	Case-control	Single	NR	121
Zhang et al. [36]	2018	USA	Surface Wave Elastography	Observational prospective	Single	NR	52
Clay et al. [12]	2019	USA	Surface Wave Elastography	Case-control	Single	NR	96
Zhou et al. [37]	2019	USA	Surface Wave Elastography	Case-control	Single	February 2016–May 2017	118
Huang et al. [38]	2022	China	SWE	Case-control	Single	March 2019–November 2020	125

Abbreviations: SWE = shear wave elastography; NR = not reported.

**Table 6:** Study regarding procedural guidance.

Author	Year	Country	Elastography Method	Study Design	Center	Inclusion Period	Sample Size
Deng et al. [39]	2025	China	SWE	Randomized Controlled Trial 1:1	Multi	April 2023–August 2024	232

Abbreviations: SWE = shear wave elastography.

**Table 7:** Studies regarding COPD, pulmonary edema, pneumothorax, and healthy aerated lung tissue.

Author	Year	Country	Elastography Method	Study Design	Center	Inclusion Period	Sample Size
Ventilation							
Girard et al. [40]	2022	Canada	SE and Surface Wave Elastography	Single-blind randomized crossover proof of concept study	Single	July 2017–October 2017	10
Pneumothorax							
Bandelli et al. [41]	2020	Italy	SE	Diagnostic accuracy	Single	January 2017– December 2018	30
COPD							
Nouvenne et al. [42]	2022	Italy	SE	Cross-sectional, pragmatic diagnostic	Single	NR	60
Cardiogenic pulmonary edema							
Wiley et al. [43]	2021	USA	Surface Wave Elastography	Observational	Single	NR	14
Healthy aerated lung tissue							
Zhang et al. [44]	2010	USA	Surface Wave Elastography	Observational	Single	NR	1

Abbreviations: SE = strain elastography; NR = not reported; COPD = chronic obstructive pulmonary disease.

**Table 8:** Studies regarding endobronchial elastography.

Author	Year	Country	Elastography Method	Study Design	Center	Inclusion Period	Sample Size
Hai-Yan et al. [45]	2017	China	7.5 MHz Convex probe EBUS (CP-EBUS; EB-1970UK, Pentax, Tokyo, Japan). Ultrasound processor HI VISION AVIUS (HITACHI, Tokyo, Japan).	Observational	Single	January 2014–October 2015	57
Zhi et al. [21]	2020	China	10 MHz EBUS (BF-UC260FW) and processor (EU-ME2) Olympus, Tokyo, Japan	Observational	Single	July 2018–December 2019	116

## Data Availability

No new data were created or analyzed in this study. The original contributions presented in this study are included in the article. Further inquiries can be directed to the corresponding author.

## Supplementary Materials

The following supporting information can be downloaded at https://www.mdpi.com/article/10.3390/cancers18020190/s1. Figure S1: Assessment of risk of bias; Figure S2: Applicability of studies; Table S1: PRISMA checklist. Reference [46] is cited in the supplementary materials.
